# Effect of standardized ileal digestible arginine:lysine on growth performance of 6- to 13-kg nursery pigs

**DOI:** 10.1093/jas/skae226

**Published:** 2024-08-08

**Authors:** Dalton C Humphrey, Chloe S Hagen, Caitlyn M Wileman, Margaret C Putnam, Keith D Haydon, Laura L Greiner

**Affiliations:** Department of Animal Science, Iowa State University, Ames, IA 50011, USA; Department of Animal Science, Iowa State University, Ames, IA 50011, USA; Department of Animal Science, Iowa State University, Ames, IA 50011, USA; Department of Animal Science, Iowa State University, Ames, IA 50011, USA; CJ America – Bio, Fort Dodge, IA 50501, USA; Department of Animal Science, Iowa State University, Ames, IA 50011, USA

**Keywords:** amino acids, arginine, swine, weaning

## Abstract

A total of 480 newly weaned pigs (PIC 337 × 1050; Genus, Hendersonville, TN) with an initial body weight (**BW**) of 6.20 ± 0.61 kg were used in a dose–response study to investigate the impact of increasing standardized ileal digestible (**SID**) Arg:Lys on nursery pig growth performance. At weaning, pigs were placed into 48 pens with 5 barrows and 5 gilts per pen. Pens were randomly assigned to 1 of 6 dietary treatments. The experimental diets were formulated with increasing SID Arg:Lys, achieved by substituting corn starch, glycine, and l-alanine with l-arginine, resulting in SID Arg:Lys ranging from 45% to 145%. Diets were sublimiting in SID Lys and exceeded all other essential amino acid requirements. The experimental diets were fed across two feeding phases from days 0 to 10 and 10 to 27, with adjustments made to account for the Lys requirement of the pigs. All pens were placed on a common diet for the remaining 14 d of the study to evaluate carryover effects. Pigs and feeders were weighed at the start and end of each phase to calculate average daily gain (**ADG**), average daily feed intake (**ADFI**), and feed efficiency (**G:F**). Data were analyzed according to a linear regression model, which included the linear and quadratic effects of SID Arg:Lys and initial BW. Pen was the experimental unit, and results were considered significant at *P* ≤ 0.05 and a tendency at 0.50 < *P* ≤ 0.10. From days 0 to 27, Arg:Lys tended to have a quadratic effect on ADFI (*P* = 0.058), where 97.00 ± 7.631% SID Arg:Lys maximized feed intake. Similarly, Arg:Lys had a quadratic impact on ADG (*P* = 0.046), where ADG was maximized at a SID Arg:Lys of 95.65 ± 7.165. Correspondingly, Arg:Lys had a quadratic effect on pig BW on day 27 (*P* = 0.014). These effects were carried through the end of the study, where Arg:Lys quadratically impacted days 0 to 41 ADFI (*P* = 0.006), ADG (*P* = 0.077), and day 41 BW (*P* = 0.028). There was no evidence of an effect of SID Arg:Lys on G:F throughout the study (*P* ≥ 0.315). In conclusion, SID Arg:Lys quadratically impacted ADFI and ADG in 6- to 13-kg nursery pigs, where ADFI was maximized at a SID Arg:Lys of 97.00% (95% CI [81.6%, 112.4%]), and ADG was maximized at a SID Arg:Lys of 95.65% (95% CI [81.2%, 110.1%]). Together, these data suggest that the SID Arg:Lys requirement of nursery pigs is at least 81%, based on the lower bounds of the 95% CI for maximum ADG and ADFI, and excessive Arg supplementation may negatively affect growth performance.

## Teaser

Feeding SID Arg:Lys levels higher than the NRC (2012) estimated requirement results in improved growth performance in nursery pigs.

## Introduction

Arginine is considered a conditionally essential amino acid (**EAA**) in swine, and it is typically assumed that, under most circumstances, dietary intake and endogenous synthesis are sufficient to meet requirements for growth and other biological functions (NRC, 2012). In swine, Arg is produced in the liver as a constituent of the urea cycle; however, this pathway does not result in a net flux of Arg to extrahepatic tissues due to the high level of Arg hydrolysis by cytosolic arginase (Wu and Morris, 1998). Therefore, endogenous synthesis relies predominantly on the intestinal-renal axis, where citrulline produced in the enterocytes of the small intestine is converted to Arg, primarily in the kidney, via arginosuccinate synthetase and arginosuccinate lyase (Brosnan and Brosnan, 2004). It has previously been suggested that Arg is an EAA in neonatal pigs due to its deficiency in sow milk, but a non-EAA in postweaned pigs (Wu et al., 2007b). However, if dietary Arg intake is marginal, endogenous synthesis may be insufficient for optimal growth.

As swine nutritionists continue to be more precise with AA nutrition, crystalline forms of the major EAA (Lys, Thr, Trp, Met, Ile, Val) are commonly included in diets, resulting in not only a reduction in dietary crude protein but also levels of non-supplemented AAs that may not be considered in formulation, which is commonly the case for conditionally EAAs, such as Arg. The NRC (2012) estimate of the standardized ileal digestible (SID) arginine requirement of 5 to 7 and 7 to 11 kg pigs is 1.8 and 2.9 g/d, respectively, which corresponds to a SID Arg:Lys of 45% and 46%. These estimates are based on a series of seminal experiments conducted by Southern and Baker (1983), who observed linear improvements in body weight (**BW**) gain in pigs fed up to 0.48% bioavailable Arg (7.62 g/kg gain) but no further improvements in growth rate at levels up to 0.68%. The SID Arg:Lys requirement, based on NRC (2012), is significantly lower than that in practical swine diets; therefore, Arg is typically disregarded in diet formulation.

Several experiments have shown benefits to supplementing Arg above NRC (2012) recommendations in nursery pigs. While some authors report improvements in feed intake and growth rate, others report benefits in feed efficiency and growth rate with no impacts on feed intake (Yao et al., 2011; Yun et al., 2020; Greiner et al., 2023; Perez-Palencia et al., 2024). On the other hand, there have also been studies that have shown negative impacts of excess Arg on pig performance (Southern and Baker, 1982; Hagemeier et al., 1983; Anderson et al., 1984; Greiner et al., 2023). However, to the authors’ knowledge, no work has been conducted to understand the SID Arg requirement relative to Lys in young pigs. The hypothesis was that feeding SID Arg:Lys levels higher than NRC (2012) estimates would improve growth performance in nursery pigs. Therefore, the objective of this study was to determine the optimal dietary SID Arg:Lys to maximize growth performance in 6- to 13-kg nursery pigs.

## Materials and Methods

### General

All experimental protocols adhered to guidelines for the ethical and humane use of animals for research according to the Guide for the Care and Use of Agricultural Animals in Research and Teaching (FASS, 2010) and were approved by the Institutional Animal Care and Use Committee at Iowa State University (IACUC 23-103).

### Animals, housing, and experimental design

The experiment was conducted at the Iowa State Swine Nutrition Farm (Ames, IA). Four hundred eighty newly weaned pigs (PIC 337 × 1050, PIC Genus, Hendersonville, TN) with an initial BW of 6.2 ± 0.61 kg were randomly placed into 48 pens with five barrows and five gilts per pen. The pens (1.21 × 2.41 m) were equipped with one 4-space feeder and two nipple waters, which ensured ad libitum access to feed and water for the duration of the study. The pigs were porcine reproductive and respiratory virus and *Mycoplasma hyopneumoniae* stable and influenza A virus and porcine epidemic diarrhea virus negative.

Upon placement, pens were randomly assigned to one of six dietary treatments (*n* = 8) according to a completely randomized design. Pigs and feeders were weighed on days 0, 10, 27, and 41 of the trial to calculate average daily gain (**ADG**), average daily feed intake (**ADFI**), and feed efficiency (**G:F**).

### Experimental diets

The experimental diets were formulated in a dose titration with increasing SID Arg:Lys by replacing corn starch, glycine, and l-alanine with l-arginine. The SID Arg:Lys levels of the experimental diets were equally spaced and ranged from 45% to 145%. The lowest SID Arg:Lys level evaluated corresponded to the NRC (2012) estimated requirement. The experimental diets were also supplemented with l-lysine HCl, l-phenylalanine, l-threonine, l-tyrosine, l-methionine, l-valine, l-histidine HCl, l-isoleucine, and l-tryptophan. In order to appropriately evaluate arginine levels relative to lysine, the diets were formulated to be sublimiting in SID Lys and exceed NRC (2012) and genetic supplier recommendations for all other EAAs (Boisen, 2003). Additionally, diets were formulated to be isocaloric and isonitrogenous through the inclusion of glycine, l-alanine, and cornstarch, and vitamin and mineral levels met or exceeded NRC (2012) recommendations.

The experimental diets were fed in two feeding phases, with phase one diets (Table 1) being fed from day 0 to 10 and phase two diets (Table 2) being fed from days 10 to 27. The same SID Arg:Lys levels were maintained across phases one and two, with Lys levels being adjusted according to the pigs’ requirements. Following phases 1 and 2, all pigs were placed on a common diet (Table 3) for the remainder of the study (days 27 to 41) to evaluate the carryover effects of the experimental diets through the end of the nursery period. Diets were manufactured at the Iowa State Swine Nutrition Farm feed mill. Feed samples for each phase and dietary treatment were collected after mixing and stored at −20 °C until analysis.

**Table 1.** Ingredient and nutrient composition of phase 1 (days 0 to 10) experimental diets

	SID Arg:Lys, %					
Ingredient	45	65	85	105	125	145
Corn	41.32	41.32	41.32	41.32	41.32	41.32
Oat groats	15.00	15.00	15.00	15.00	15.00	15.00
Dried whey	11.94	11.94	11.94	11.94	11.94	11.94
Corn gluten meal	9.12	9.12	9.12	9.12	9.12	9.12
Soybean meal 47.5% CP	6.26	6.26	6.26	6.26	6.26	6.26
Soybean oil	5.32	5.32	5.32	5.32	5.32	5.32
Monocalcium phosphate	2.00	2.00	2.00	2.00	2.00	2.00
l-Alanine	1.35	1.08	0.81	0.54	0.27	0.00
Glycine	1.14	0.91	0.68	0.46	0.23	0.00
l-Lysine HCl	1.10	1.10	1.10	1.10	1.10	1.10
Calcium carbonate	1.06	1.06	1.06	1.06	1.06	1.06
l-Phenylalanine	0.52	0.52	0.52	0.52	0.52	0.52
Sodium chloride	0.50	0.50	0.50	0.50	0.50	0.50
l-Threonine	0.49	0.49	0.49	0.49	0.49	0.49
l-Tyrosine	0.47	0.47	0.47	0.47	0.47	0.47
Zinc oxide	0.38	0.38	0.38	0.38	0.38	0.38
l-Methionine	0.37	0.37	0.37	0.37	0.37	0.37
VTM premix^1^	0.35	0.35	0.35	0.35	0.35	0.35
l-Valine	0.35	0.35	0.35	0.35	0.35	0.35
l-Histidine HCl	0.23	0.23	0.23	0.23	0.23	0.23
l-Isoleucine	0.21	0.21	0.21	0.21	0.21	0.21
l-Tryptophan	0.17	0.17	0.17	0.17	0.17	0.17
Copper sulfate	0.08	0.08	0.08	0.08	0.08	0.08
Potassium carbonate	0.09	0.09	0.09	0.09	0.09	0.09
Sodium bicarbonate	0.19	0.19	0.19	0.19	0.19	0.19
Corn starch	0.00	0.23	0.47	0.70	0.93	1.17
l-Arginine	0.00	0.26	0.53	0.79	1.06	1.32
Total	100.00	100.00	100.00	100.00	100.00	100.00
Calculated composition						
ME, Mcal/kg	3.40	3.40	3.40	3.40	3.40	3.40
Crude protein, %	20.31	20.31	20.31	20.31	20.32	20.32
Total calcium, %	0.85	0.85	0.85	0.85	0.85	0.85
Available phosphorus, %	0.55	0.55	0.55	0.55	0.55	0.55
dEB, meq/kg	100.75	100.75	100.75	100.75	100.75	100.75
SID Lys, %	1.30	1.30	1.30	1.30	1.30	1.30
SID Arg, %	0.59	0.85	1.11	1.37	1.63	1.89
SID Arg:Lys	0.45	0.65	0.85	1.05	1.25	1.45
SID His:Lys	0.35	0.35	0.35	0.35	0.35	0.35
SID Ile:Lys	0.54	0.54	0.54	0.54	0.54	0.54
SID Leu:Lys	1.22	1.22	1.22	1.22	1.22	1.22
SID Met + Cys:Lys	0.64	0.64	0.64	0.64	0.64	0.64
SID Phe:Lys	0.69	0.69	0.69	0.69	0.69	0.69
SID Tyr:Lys	0.52	0.52	0.52	0.52	0.52	0.52
SID Thr:Lys	0.72	0.72	0.72	0.72	0.72	0.72
SID Trp:Lys	0.22	0.22	0.22	0.22	0.22	0.22
SID Val:Lys	0.70	0.70	0.70	0.70	0.70	0.70
Analyzed composition						
Crude protein, %	20.01	19.69	20.96	21.52	20.82	20.21
Total Lys, %	1.58	1.43	1.52	1.49	1.41	1.29
Total Arg, %	0.72	0.92	1.19	1.49	1.66	1.85
Total His, %	0.51	0.52	0.51	0.52	0.49	0.57
Total Ile, %	0.92	0.82	0.89	0.91	0.89	0.80
Total Leu, %	1.90	1.67	1.87	1.82	1.76	1.80
Total Met + Cys, %	1.00	0.89	0.82	0.89	0.83	0.88
Total Phe, %	1.30	1.26	1.29	1.34	1.30	1.29
Total Tyr, %	0.93	0.91	0.91	0.96	0.93	0.92
Total Thr, %	1.26	0.96	1.30	0.94	0.88	0.94
Total Trp, %	0.27	0.27	0.27	0.27	0.28	0.26
Total Val, %	1.07	1.07	1.07	1.12	1.04	1.08

^1^Vitamin trace mineral premix; provided 4,594 IU vitamin A, 525 IU vitamin D, 37.5 IU vitamin E, 2.25 mg vitamin K, 8.25 mg riboflavin, 42 mg niacin, 20.25 mg pantothenic acid, 0.04 mg vitamin B12, 12 mg Cu (copper sulfate), 0.28 mg I (potassium iodate), 160 mg Fe (ferrous sulfate), 0.30 mg Se (sodium selenate), and 160 mg Zn (zinc sulfate) per kilogram of the diet.

**Table 2.** Ingredient and nutrient composition of phase 2 (days 10 to 27) experimental diets

	SID Arg:Lys, %					
Ingredient	45	65	85	105	125	145
Corn	56.55	56.55	56.55	56.55	56.55	56.55
Corn gluten meal	9.76	9.76	9.76	9.76	9.76	9.76
Soybean meal 47.5% CP	6.21	6.21	6.21	6.21	6.21	6.21
Dried whey	5.97	5.97	5.97	5.97	5.97	5.97
Soybean oil	5.22	5.22	5.22	5.22	5.22	5.22
Oat groats	5.00	5.00	5.00	5.00	5.00	5.00
Monocalcium phosphate	2.19	2.19	2.19	2.19	2.19	2.19
l-Alanine	1.30	1.04	0.78	0.52	0.26	0.00
Calcium carbonate	1.11	1.11	1.11	1.11	1.11	1.11
l-Lysine HCl	1.10	1.10	1.10	1.10	1.10	1.10
Glycine	1.09	0.88	0.66	0.44	0.22	0.00
Sodium chloride	0.50	0.50	0.50	0.50	0.50	0.50
l-Phenylalanine	0.50	0.50	0.50	0.50	0.50	0.50
l-Threonine	0.49	0.49	0.49	0.49	0.49	0.49
l-Tyrosine	0.48	0.48	0.48	0.48	0.48	0.48
Zinc oxide	0.38	0.38	0.38	0.38	0.38	0.38
VTM premix^1^	0.35	0.35	0.35	0.35	0.35	0.35
l-Methionine	0.35	0.35	0.35	0.35	0.35	0.35
l-Valine	0.34	0.34	0.34	0.34	0.34	0.34
l-Histidine HCl	0.20	0.20	0.20	0.20	0.20	0.20
l-Isoleucine	0.19	0.19	0.19	0.19	0.19	0.19
l-Tryptophan	0.18	0.18	0.18	0.18	0.18	0.18
Copper sulfate	0.08	0.08	0.08	0.08	0.08	0.08
Potassium carbonate	0.16	0.16	0.16	0.16	0.16	0.16
Sodium bicarbonate	0.33	0.33	0.33	0.33	0.33	0.33
Corn starch	0.00	0.23	0.45	0.67	0.90	1.12
l-Arginine	0.00	0.25	0.51	0.76	1.02	1.27
Total	100.00	100.00	100.00	100.00	100.00	100.00
Calculated composition						
ME, Mcal/kg	3.40	3.40	3.40	3.40	3.40	3.40
Crude protein, %	19.53	19.53	19.53	19.53	19.53	19.53
Total calcium, %	0.85	0.85	0.85	0.85	0.85	0.85
Available phosphorus, %	0.55	0.55	0.55	0.55	0.55	0.55
dEB, meq/kg	100.00	100.00	100.00	100.00	100.00	100.00
SID Lys, %	1.25	1.25	1.25	1.25	1.25	1.25
SID Arg, %	0.56	0.81	1.06	1.31	1.56	1.81
SID Arg:Lys	0.45	0.65	0.85	1.05	1.25	1.45
SID His:Lys	0.35	0.35	0.35	0.35	0.35	0.35
SID Ile:Lys	0.52	0.52	0.52	0.52	0.52	0.52
SID Leu:Lys	1.30	1.30	1.30	1.30	1.30	1.30
SID Met + Cys:Lys	0.64	0.64	0.64	0.64	0.64	0.64
SID Phe:Lys	0.69	0.69	0.69	0.69	0.69	0.69
SID Tyr:Lys	0.52	0.52	0.52	0.52	0.52	0.52
SID Thr:Lys	0.72	0.72	0.72	0.72	0.72	0.72
SID Trp:Lys	0.22	0.22	0.22	0.22	0.22	0.22
SID Val:Lys	0.70	0.70	0.70	0.70	0.70	0.70
Analyzed composition						
Crude protein, %	19.01	19.57	19.83	19.37	19.54	19.98
Total Lys, %	1.38	1.33	1.32	1.42	1.34	1.39
Total Arg, %	0.74	0.86	1.08	1.34	1.55	1.72
Total His, %	0.53	0.53	0.53	0.52	0.52	0.55
Total Ile, %	0.83	0.80	0.73	0.85	0.79	0.84
Total Leu, %	1.99	1.88	1.75	1.89	1.82	1.86
Total Met + Cys, %	0.87	0.78	0.72	0.86	0.81	0.94
Total Phe, %	1.31	1.21	1.18	1.25	1.25	1.27
Total Tyr, %	0.98	0.90	0.88	0.93	0.93	0.86
Total Thr, %	0.99	0.90	0.84	1.03	0.97	0.92
Total Trp, %	0.22	0.24	0.26	0.26	0.26	0.25
Total Val, %	1.04	0.96	0.98	1.05	1.00	1.05

^1^Vitamin trace mineral premix; provided 4,594 IU vitamin A, 525 IU vitamin D, 37.5 IU vitamin E, 2.25 mg vitamin K, 8.25 mg riboflavin, 42 mg niacin, 20.25 mg pantothenic acid, 0.04 mg vitamin B12, 12 mg Cu (copper sulfate), 0.28 mg I (potassium iodate), 160 mg Fe (ferrous sulfate), 0.30 mg Se (sodium selenate), and 160 mg Zn (zinc sulfate) per kilogram of the diet.

**Table 3.** Ingredient and nutrient composition of phase 3 (days 27 to 41) common diet

Ingredient	Inclusion, %
Corn	60.68
Soybean meal 47.5% CP	32.00
Soybean oil	3.59
Monocalcium phosphate	0.99
Calcium carbonate	0.62
l-Lysine HCl	0.51
Sodium chloride	0.50
VTM premix^1^	0.35
l-Methionine	0.27
l-Threonine	0.21
l-Valine	0.19
l-Tryptophan	0.05
l-Isoleucine	0.04
Phytase^2^	0.01
Total	100.00
Calculated composition	
ME, Mcal/kg	3.40
Crude protein, %	20.38
Total calcium, %	0.65
Available phosphorus, %	0.40
dEB, meq/kg	172.45
SID Lys, %	1.33
SID Arg, %	1.21
SID Arg:Lys	0.91
SID His:Lys	0.33
SID Ile:Lys	0.55
SID Leu:Lys	1.04
SID Met + Cys:Lys	0.58
SID Phe:Lys	0.64
SID Tyr:Lys	0.33
SID Thr:Lys	0.60
SID Trp:Lys	0.19
SID Val:Lys	0.70
Analyzed composition	
Crude protein, %	20.18
Total Lys, %	1.53
Total Arg, %	1.34
Total His, %	0.56
Total Ile, %	0.96
Total Leu, %	1.70
Total Met + Cys, %	0.81
Total Phe, %	1.02
Total Tyr, %	0.68
Total Thr, %	0.89
Total Trp, %	0.26
Total Val, %	1.17

^1^Vitamin trace mineral premix; provided 4,594 IU vitamin A, 525 IU vitamin D, 37.5 IU vitamin E, 2.25 mg vitamin K, 8.25 mg riboflavin, 42 mg niacin, 20.25 mg pantothenic acid, 0.04 mg vitamin B12, 12 mg Cu (copper sulfate), 0.28 mg I (potassium iodate), 160 mg Fe (ferrous sulfate), 0.30 mg Se (sodium selenate), and 160 mg Zn (zinc sulfate) per kilogram of the diet.

^2^Phytase activity 450 FTU/kg.

### Diet analysis

Feed samples were ground through a 1 mm screen using a Wiley Mill (Variable Speed Digital ED-5 Wiley Mill; Thomas Scientific, Swedesboro, NJ). Ground samples were submitted to the University of Missouri Agricultural Experimental Station Laboratories (Columbia, MO) for complete AA profiling using cation-exchange chromatography coupled with post-column ninhydrin derivatization and quantification (method 982.30 E and 988.15; AOAC, 2006).

### Statistical analysis

Growth performance data were analyzed according to the following linear model:


$$\begin{array}{l} y_{i}=\beta_{0}+\beta_{1}x_{i1}+\beta_{2}x_{i1}^{2}+\beta_{3}x_{i2}+\varepsilon_{i} \end{array}$$

Where *y*_*i*_ is the response (ADG, ADFI, G:F) of the *i*th pen, $\beta_{0}$ is the intercept term, $\beta_{1}$ is the estimated linear coefficient for SID Arg:Lys, $\beta_{2}$ is the estimated quadratic coefficient for SID Arg:Lys, $\beta_{3}$ is the estimated coefficient for initial BW, and $\varepsilon_{i}$ is the random error associated with $y_{i}$, assuming $\varepsilon_{i}∼N\left(0,I\sigma_{\varepsilon }^{2}\right).$ Linear-plateau and quadratic-plateau models were also evaluated; however, due to the quadratic nature of the response, these models did not converge. The linear regression models were fit using the lm function in R version 4.3.1 (R Core Team, 2023). Homogeneity of variance was evaluated using the Breusch–Pagen Test using the ncvTest function of the car package (v3.1.2; Fox and Weisberg, 2019). Studentized Residuals were calculated using the studres() function from the MASS package (v7.3.60.2; Venables and Ripley, 2002). Normality of the Studentized Residuals was evaluated using the Shapiro–Wilk test. Validity of the assumptions of the models was further verified through analysis of residual diagnostic plots. Studentized Residuals greater than approximately 3 standard deviations from the mean were considered statistical outliers and excluded from the analysis. Model fit was assessed using the omnibus *F*-test. Additionally, root mean squared error and *R*^2^ were calculated for all models. Pen was the experimental unit, and parameters were considered significant if *P* ≤ 0.05 and a tendency at 0.05 < *P* ≤ 0.10. Plots of the regression curves were constructed using the ggplot package (v3.5.2; Wickham, 2016).

For models where the quadratic coefficient *P* ≤ 0.10, the SID Arg:Lys level that maximized the regression curve (Arg:Lys_max_) was calculated through differentiation with respect to SID Arg:Lys, yielding the following equation:


$$\begin{array}{l} SID\;\;\;Arg:Lys_{max}=\frac{-\beta_{1}}{2\beta_{2}} \end{array}$$

The variance and corresponding standard error and 95% confidence intervals for $SID\;\;\;Arg:Lys_{max}$ were then calculated using the Delta Method.

## Results

The analyzed total AA and crude protein (**CP**) concentrations of the diets (Tables 1, 2, and 3 for phases 1, 2, and 3, respectively) were consistent with calculated values based on normal variation (Cromwell et al., 1999). Specifically, Arg:Lys increased across the experimental diets, while CP concentration remained similar across diets within a phase.

The pigs started the study at an initial BW of 6.20 ± 0.611 kg and ended the study on day 41 at 21.17 ± 1.864 kg. Based on the observed data (Table 4), pigs fed diets containing 45% or 145% SID Arg:Lys had numerically the lowest mean BW, ADG, and ADFI when the experimental diets were fed (days 0 to 27) and over the entire study (days 0 to 41), while pigs fed SID Arg:Lys between 65% and 125% appeared to perform more similarly.

**Table 4.** Observed performance of nursery pigs fed increasing levels of SID Arg:Lys^1^

	Dietary SID Arg:Lys, %^2^					
Item	45	65	85	105	125	145
BW day 0, kg	6.26 (0.617)	6.27 (0.730)	6.41 (0.807)	6.02 (0.557)	6.25 (0.577)	6.01 (0.403)
Experimental diet period (days 0 to 27)						
BW day 27, kg	12.36 (1.692)	13.18 (1.593)	13.13 (1.513)	13.01 (1.027)	12.87 (1.062)	12.17 (0.896)
ADG, kg	0.22 (0.046)	0.25 (0.039)	0.24 (0.027)	0.24 (0.026)	0.24 (0.024)	0.22 (0.032)
ADFI, kg	0.31 (0.057)	0.35 (0.062)	0.34 (0.040)	0.34 (0.033)	0.33 (0.028)	0.31 (0.034)
G:F	0.70 (0.036)	0.72 (0.033)	0.71 (0.019)	0.72 (0.018)	0.73 (0.043)	0.70 (0.072)
SID Arg intake, g/d	1.79 (0.320)	2.86 (0.504)	3.65 (0.427)	4.55 (0.408)	5.25 (0.424)	5.78 (0.629)
Common diet period (days 27 to 41)						
BW day 41, kg	20.32 (2.483)	21.59 (2.360)	21.22 (1.595)	22.04 (1.469)	21.45 (1.574)	20.42 (1.333)
ADG, kg	0.57 (0.074)	0.60 (0.074)	0.57 (0.051)	0.63 (0.034)	0.61 (0.045)	0.58 (0.064)
ADFI, kg	0.84 (0.084)	0.85 (0.116)	0.85 (0.065)	0.91 (0.041)	0.86 (0.057)	0.82 (0.053)
G:F	0.68 (0.051)	0.70 (0.041)	0.67 (0.049)	0.70 (0.034)	0.71 (0.014)	0.70 (0.067)
Overall period (days 0 to 41)						
ADG, kg	0.34 (0.053)	0.37 (0.046)	0.35 (0.024)	0.37 (0.028)	0.36 (0.033)	0.33 (0.025)
ADFI, kg	0.49 (0.063)	0.52 (0.070)	0.51 (0.034)	0.53 (0.038)	0.51 (0.037)	0.48 (0.029)
G:F	0.69 (0.037)	0.71 (0.030)	0.69 (0.027)	0.70 (0.016)	0.71 (0.024)	0.70 (0.042)

^1^A total of 480 pigs across 48 pens (10 pigs/pen) with eight pens per treatment.

^2^Data reported as observed mean (standard deviation).

Table 5 presents the predicted means and standard errors for the performance of nursery pigs in response to the SID Arg:Lys levels evaluated in the current study based on the fitted quadratic regression models, adjusting to the average starting BW across dietary treatments (6.20 kg). Therefore, these values represent the expected response to increasing SID Arg:Lys after accounting for the variation due to initial BW. The predicted performance values numerically show similar trends as those exhibited in the observed data, although the amount of variation captured by the regression models, as indicated by the various model fit statistics presented in Table 6, varied across response variables.

**Table 5.** Predicted performance of nursery pigs fed increasing levels of SID Arg:Lys estimated from the fitted quadratic regression models^1^

	Dietary SID Arg:Lys, %^2^^,^^3^						*P* value		
Item	45	65	85	105	125	145	Arg:Lys	Arg:Lys^2^	Initial BW
BW day 0, kg	6.26 (0.198)	6.28 (0.121)	6.27 (0.133)	6.22 (0.133)	6.14 (0.121)	6.03 (0.198)	0.373	0.637	–
Experimental diet period (days 0 to 27)									
BW day 27, kg	12.35 (0.244)	12.82 (0.150)	13.07 (0.164)	13.09 (0.164)	12.89 (0.149)	12.47 (0.246)	0.718	0.014	<0.001
ADG, kg	0.22 (0.009)	0.24 (0.005)	0.24 (0.006)	0.24 (0.006)	0.24 (0.005)	0.22 (0.009)	0.928	0.046	<0.001
ADFI, kg	0.32 (0.011)	0.33 (0.007)	0.34 (0.008)	0.34 (0.007)	0.33 (0.007)	0.32 (0.011)	0.792	0.058	<0.001
G:F	0.71 (0.010)	0.71 (0.006)	0.72 (0.007)	0.72 (0.006)	0.71 (0.006)	0.71 (0.011)	0.976	0.315	0.676
SID Arg intake, g/d	1.75 (0.110)	2.80 (0.068)	3.72 (0.074)	4.52 (0.074)	5.19 (0.069)	5.73 (0.117)	<0.001	0.003	<0.001
Common diet period (days 27 to 41)									
BW day 41, kg	20.25 (0.459)	21.12 (0.282)	21.62 (0.309)	21.73 (0.308)	21.46 (0.281)	20.82 (0.462)	0.358	0.028	<0.001
ADG, kg	0.57 (0.020)	0.59 (0.012)	0.60 (0.013)	0.61 (0.013)	0.60 (0.012)	0.58 (0.020)	0.485	0.149	0.557
ADFI, kg	0.82 (0.020)	0.86 (0.012)	0.89 (0.013)	0.89 (0.013)	0.87 (0.012)	0.83 (0.019)	0.791	0.005	0.127
G:F	0.68 (0.013)	0.68 (0.008)	0.69 (0.009)	0.69 (0.009)	0.70 (0.008)	0.71 (0.014)	0.103	0.605	0.195
Overall period (days 0 to 41)									
ADG, kg	0.34 (0.011)	0.36 (0.007)	0.36 (0.007)	0.36 (0.007)	0.36 (0.007)	0.35 (0.011)	0.751	0.077	0.007
ADFI, kg	0.48 (0.012)	0.50 (0.007)	0.52 (0.008)	0.52 (0.008)	0.51 (0.007)	0.49 (0.012)	0.637	0.006	<0.001
G:F	0.69 (0.010)	0.70 (0.006)	0.70 (0.007)	0.70 (0.007)	0.70 (0.006)	0.70 (0.010)	0.521	0.482	0.290

^1^A total of 480 pigs across 48 pens (10 pigs/pen) with eight pens per treatment.

^2^Data reported as predicted mean (standard error); SID Arg intake evaluated using calculated diet SID Arg:Lys values.

^3^Initial BW in the regression models was set at the mean (6.20 kg).

**Table 6.** Model fit statistics and calculated maximum of quadratic regression curves

			Omnibus *F*-test				SID Arg:Lys_max_ 95% CI	
Item	*R* ^2^	RMSE	*F*-statistic	*P* value	SID Arg:Lys_max_^1^	S.E.	Lower limit	Upper limit
BW day 0, kg	0.023	0.598	0.52	0.599	–	–	–	–
Experimental diet period (day 0 to 27)								
BW day 27, kg	0.690	0.728	32.59	<0.001	97.09	5.834	85.34	108.85
ADG, kg	0.371	0.026	8.63	<0.001	95.65	7.165	81.21	110.09
ADFI, kg	0.416	0.033	10.43	<0.001	97.00	7.632	81.62	112.39
G:F	0.027	0.029	0.39	0.759	–	–	–	–
Common diet period (days 27 to 41)								
BW day 41, kg	0.449	1.369	11.94	<0.001	101.00	6.986	86.92	115.07
ADG, kg	0.065	0.058	1.02	0.395	–	–	–	–
ADFI, kg	0.224	0.056	4.04	0.013	96.34	5.026	86.19	106.48
G:F	0.115	0.040	1.86	0.150	–	–	–	–
Overall period (days 0 to 41)								
ADG, kg	0.218	0.033	4.08	0.012	97.59	8.267	80.93	114.25
ADFI, kg	0.376	0.035	8.64	<0.001	97.41	5.113	87.10	107.72
G:F	0.047	0.030	0.73	0.540	–	–	–	–

^1^Calculated maximum of quadratic regression curve. Maximum of the curve was calculated when quadratic coefficient *P* ≤ 0.1.

**Figure 1.**
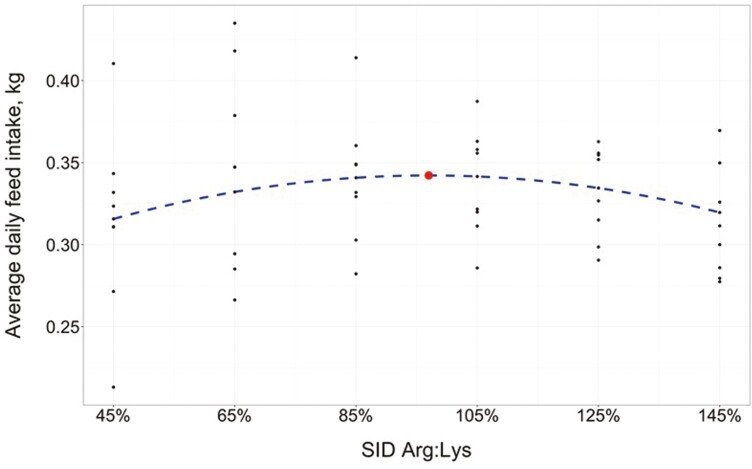
Fitted quadratic regression model on ADFI in response to increasing SID Arg:Lys in 6- to 13-kg (days 0 to 27) nursery pigs (*R*^2^ = 0.416). Maximum ADFI (indicated by the circle) was estimated at 97.00% (95% CI: [81.6%, 112.4%]) SID Arg:Lys. Initial BW in the model was set at the mean (6.20 kg).


From days 0 to 27, there was a tendency for a quadratic effect of SID Arg:Lys on ADFI (*P* = 0.058; Table 5), where predicted ADFI was maximized at a SID Arg:Lys of 97.00 ± 7.631% (95% CI: [81.6%, 112.4%]; Table 6). The estimated regression equation for ADFI (days 0 to 27; Figure 1) was

$$\begin{array}{lll} ADFI,\;kg/d= & -0.0139+0.1901(Arg:Lys)-0.0980{\left(Arg:Lys\right)}^{2}\; & +0.0426(initial\;BW,\;kg) \end{array}$$

**Figure 2.**
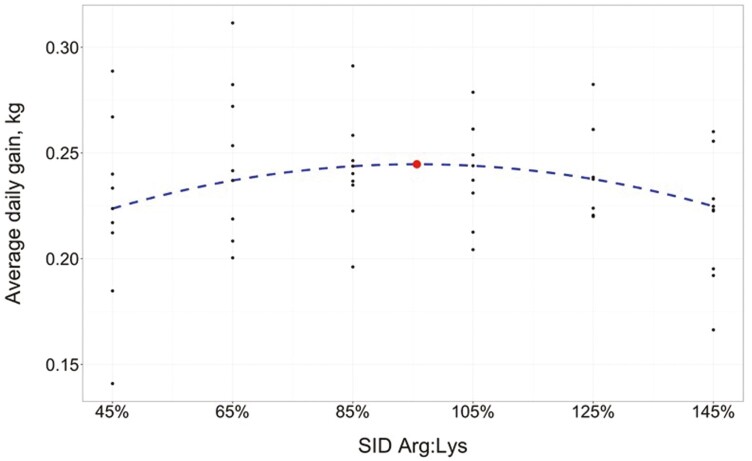
Fitted quadratic regression model on ADG in response to increasing SID Arg:Lys in 6- to 13-kg (days 0 to 27) nursery pigs (*R*^2^ = 0.371). Maximum ADG (indicated by the circle) was estimated at 95.65% (95% CI: [81.21%, 110.09%]) SID Arg:Lys. Initial BW in the model was set at the mean (6.20 kg).


Where the SID Arg:Lys in the equation is expressed as a proportion (i.e., 0.45) rather than a percentage. This trend continued through the end of the study, resulting in a significant quadratic effect of SID Arg:Lys on overall (days 0 to 41) ADFI (*P* = 0.006), which was maximized at a SID Arg:Lys of 97.41 ± 5.113% (95% CI: [87.1%, 107.7%]). Similarly, there was a quadratic effect of SID Arg:Lys on ADG from days 0 to 27 (*P* = 0.046), where predicted ADG was maximized at a SID Arg:Lys of 95.65 ± 7.165% (95% CI: [81.2%, 110.1%]). The estimated regression equation for ADG (days 0 to 27; Figure 2) was

$$\begin{array}{lll} ADG,\;kg/d= & -0.0136+0.1564(Arg:Lys)-0.0817{\left(Arg:Lys\right)}^{2}\; & +0.0296(inital\;BW,\;kg) \end{array}$$

**Figure 3.**
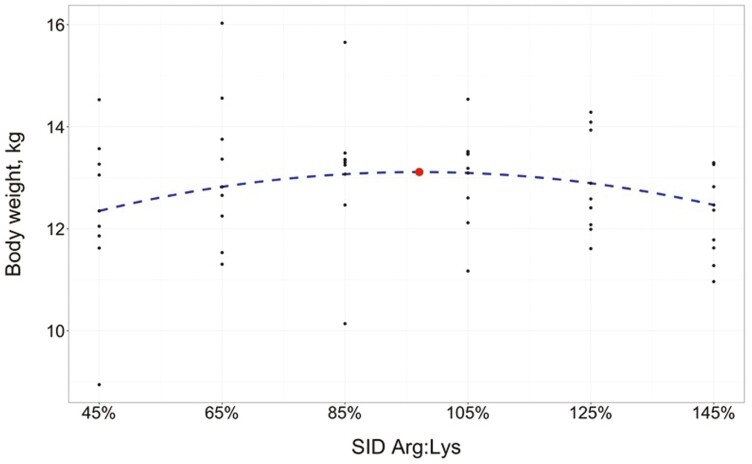
Fitted quadratic regression model on nursery pig BW on day 27 in response to increasing SID Arg:Lys (*R*^2^ = 0.690). Maximum BW (indicated by the circle) was estimated at 97.09% (95% CI: [85.34%, 108.85%]) SID Arg:Lys. Initial BW in the model was set at the mean (6.20 kg).


Additionally, there was a tendency for a quadratic effect of SID Arg:Lys on overall (days 0 to 41) ADG (*P* = 0.077), where predicted ADG was maximized at a SID Arg:Lys of 97.59 ± 8.267% (95% CI: [80.9%, 114.3%]). On day 27, there was a quadratic effect of SID Arg:Lys on pig BW (*P* = 0.014), where predicted BW was maximized at a SID Arg:Lys of 97.09 ± 5.834% (95% CI: [85.3%, 108.9%]). The estimated regression equation for BW on day 27 (Figure 3) was

$$\begin{array}{lll} BW,\;kg= & -0.1216+5.4537(Arg:Lys)-2.8084{\left(Arg:Lys\right)}^{2}\; & +1.7074(initial\;BW,\;kg) \end{array}$$

These effects were carried through the end of the study on day 41 (quadratic *P* = 0.028), where predicted BW was maximized at SID Arg:Lys of 101.00 ± 6.986% (95% CI: [86.9%, 115.1%]). There was no evidence for an effect of SID Arg:Lys on G:F through the entirety of the study (*P* ≥ 0.315).

## Discussion

The NRC (2012) classified Arg as a conditionally EAA, indicating that, under certain circumstances, the rate of Arg utilization is greater than endogenous synthesis, resulting in a dietary requirement to achieve maximal growth. The route and extent of endogenous Arg synthesis are dependent on the age of the pig. In neonatal pigs, endogenous Arg synthesis has been shown to occur primarily in the enterocytes of the small intestine through the conversion of proline and glutamine to citrulline, which is then converted to Arg via arginosuccinate synthetase and arginosuccinate lyase (Wu and Knabe, 1995; Bertolo et al., 2003; Brosnan and Brosnan, 2004). However, in postweaned pigs, arginosuccinate lyase activity is low in enterocytes (Wu and Knabe, 1995). Additionally, high arginase activity in enterocytes of postweaned pigs results in the catabolism of approximately 40% of absorbed Arg (Wu et al., 2007a). Consequently, endogenous Arg synthesis in postweaned pigs occurs primarily in the kidney from intestinal-derived citrulline, which is known as the intestinal-renal axis (Wu et al., 2018). Although pigs have the capacity for endogenous Arg synthesis, there has been little work to determine the optimal dietary level of Arg required for maximizing growth. Therefore, the current study aimed to quantify the optimal dietary SID Arg level to maximize growth in nursery pigs.

Amino acid (**AA**) requirements can be expressed in several ways, including concentration in the diet, relative to energy, relative to other AA, or relative to BW gain; however, when determining AA requirements, the experimental design and diet formulation strategy dictates how requirements can be expressed. In following the ideal protein concept, it is common to express AA requirements relative to Lys (Van Milgen and Dourmad, 2015). In doing so, Lys must be second limiting behind the AA of interest to accurately evaluate requirements relative to Lys (Boisen, 2003). Accordingly, in the current study, the SID Lys content of the diets was below NRC (2012) and genetic supplier recommendations for this weight of pig, while all other EAA were supplied above requirements, thus ensuring that Lys was second limiting. Furthermore, due to the semi-purified nature of the experimental diets, glycine, and l-alanine were supplemented in the diets as a nonspecific nitrogen source to ensure the diets were isonitrogenous and to avoid a growth response due to increased nitrogen available for non-EAA synthesis from increasing Arg in the diet.

The NRC (2012) estimated SID Arg requirement for 5- to 7- and 7- to 11-kg pigs is 1.8 (SID Arg:Lys 45%) and 2.9 g/d (SID Arg:Lys 46%), respectively. By propagating the uncertainty associated with the regression coefficients to estimate the standard error of SID Arg:Lys_max_, the results of the current study suggest, with 95% CI, the SID Arg:Lys requirement to maximize feed intake and growth rate in 6- to 13-kg pigs is between approximately 81% and 112%. To the authors’ knowledge, this is the first study that has quantified the uncertainty around the SID Arg:Lys requirement; therefore, further work is warranted to reduce the uncertainty around these estimates. Nonetheless, these results suggest that the SID Arg:Lys requirement for 6- to 13-kg pigs under the conditions of this study is approximately twice that of NRC (2012) recommendations, which is similar to the requirement proposed by Wu (2014). The NRC (2012) SID Arg requirement is based on work conducted by Southern and Baker (1983), who observed improvements in growth performance in 9- to 15-kg pigs fed up to 0.48% bioavailable Arg, but no further improvements when pigs were fed up to 0.68% bioavailable Arg. Furthermore, feeding less than 0.48% bioavailable Arg resulted in increased plasma NH_3_–N and decreased urea-N, which suggests that Arg deficiency was limiting urea biosynthesis and ammonia detoxification. However, although at least 0.48% bioavailable Arg appears to be required for optimal urea cycle function, because the levels of other EAA are not reported for these diets, the lack of growth response at higher levels of Arg may be due to limitations of other EAA. Additionally, the diets fed by Southern and Baker (1983) contained supplemental glutamic acid, which could serve as a precursor for endogenous Arg synthesis and alter the dietary requirement for Arg; however, further research is needed to understand the impacts of dietary precursors for Arg, such as glutamate, glutamine, and proline, on the dietary Arg requirement.

More recently, there have been several studies showing benefits of supplementing Arg in nursery pig diets under various conditions (Hernandez et al., 2009; Liu et al., 2009; Wu et al., 2010; Yao et al., 2011; Yun et al., 2020; Greiner et al., 2023; Perez-Palencia et al., 2024). For example, Greiner et al. (2023) fed three levels of SID Arg (1.35%, 1.55%, and 1.75%) to newly weaned pigs (5.17 kg) and observed a quadratic response in feed intake, growth rate, and feed efficiency, which were maximized at approximately 1.55% SID Arg. The response observed by Greiner et al. (2023) corresponded to 103% and 109% SID Arg:Lys from days 0 to 6 and days 6 to 20 postweaning, respectively, which is within the 95% confidence intervals for the estimated SID Arg:Lys requirement determined in the current study. Perez-Palencia et al. (2024) conducted two experiments with newly weaned pigs (5.79 and 6.11 kg in experiments 1 and 2, respectively) and fed 0.66% to 1.86% or 1.15% to 2.35% SID Arg. In the first experiment, Perez-Palencia et al. (2024) reported linear improvements in growth rate and efficiency in the first 3-wk postweaning, while in the second experiment, they observed a linear increase in feed intake and growth rate in the first week postweaning. Yao et al. (2011) supplemented 1% l-Arg in a basal diet containing 1.14% total Arg and 1.35% total Lys (135% total Arg:Lys in basal diet) and reported increased growth rate and improved feed efficiency at day 7 postweaning. However, because the objective of these previous studies focused on evaluating supplemental Arg or Arg concentration in the diet, Arg:Lys was not constant across feeding phases, diets were not isonitrogenous, and diet concentrations of other EAA were frequently not reported. Recently, Hagen et al. (2024) evaluated the response of enterically challenged nursery pigs to increasing SID Arg:Lys from 85% to 115% with diets adequate in Lys and observed linear improvements in BW at 42 d postweaning. Therefore, the response to supplemental Arg reported in the literature is largely inconsistent, which may suggest that the quantitative requirement may depend on environmental and biological factors such as the age or weight of the pig, health status, or diet composition. However, due to differences in diet formulation and experimental design, direct comparison with the current study is limited. Nonetheless, these experiments provide further evidence that feeding Arg levels higher than NRC (2012) requirement estimates lead to improved growth performance in nursery pigs.

In the current study, ADG, pig BW, and ADFI responded quadratically to increasing SID Arg:Lys in the diet, while G:F was not affected, indicating the growth response was mediated by feed intake. A reduction in feed intake in response to elevated Arg levels is consistent with early work focusing on interactions between Lys and Arg (Southern and Baker, 1982; Hagemeier et al., 1983; Anderson et al., 1984). These authors concluded that reduced performance due to excess Arg was caused by a general AA imbalance rather than an antagonism with lysine. Reduced feed intake in response to excess Arg is further supported by Greiner et al. (2023), which reported a quadratic response with 1.35% to 1.75% SID Arg. In contrast, other studies have observed no negative impact on feed intake when feeding over 2% Arg (Zhan et al., 2008; Yao et al., 2011; Zheng et al., 2013; Perez-Palencia et al., 2024). The cause of variation in the feed intake response to excess Arg is not well understood and deserves further investigation.

In conclusion, the SID Arg:Lys requirement of 6- to 13-kg nursery pigs is at least 81%, based on the lower bounds of the 95% confidence intervals for maximum ADG and ADFI; however, feeding excess levels of Arg appears to reduce performance through feed intake mediating mechanisms.

## Abbreviations

AA: amino acid
ADFI: average daily feed intake
ADG: average daily gain
BW: body weight
CP: crude protein
EAA: essential amino acid
G:F: gain-to-feed ratio
SID: standardized ileal digestible
